# Identification of subpopulations of prairie voles differentially susceptible to peer influence to decrease high alcohol intake

**DOI:** 10.3389/fphar.2013.00084

**Published:** 2013-07-04

**Authors:** Allison M. J. Anacker, Andrey E. Ryabinin

**Affiliations:** Department of Behavioral Neuroscience, Oregon Health & Science University, PortlandOR, USA

**Keywords:** prairie vole, social behavior, alcohol, ethanol, peer pressure, vasopressin, genetics, regulatory microsatellite

## Abstract

Peer influences are critical in the decrease of alcohol (ethanol) abuse and maintenance of abstinence. We previously developed an animal model of inhibitory peer influences on ethanol drinking using prairie voles and here sought to understand whether this influential behavior was due to specific changes in drinking patterns and to variation in a microsatellite sequence in the regulatory region of the vasopressin receptor 1a gene (*avpr1a*). Adult prairie voles’ drinking patterns were monitored in a lickometer apparatus that recorded each lick a subject exhibited during continuous access to water and 10% ethanol during periods of isolation, pair housing of high and low drinkers, and subsequent isolation. Analysis of fluid consumption confirmed previous results that high drinkers typically decrease ethanol intake when paired with low drinkers, but that a subset of voles do not decrease. Analysis of bout structure revealed differences in the number of ethanol drinking bouts in the subpopulations of high drinkers when paired with low drinkers. Lickometer drinking patterns analyzed by visual and by cross-correlation analyses demonstrated that pair housing did not increase the rate of subjects drinking in bouts occurring at the same time. The length of the *avpr1a* microsatellite did not predict susceptibility to peer influence or any other drinking behaviors. In summary, subpopulations of high drinkers were identified, by fluid intake and number of drinking bouts, which did or did not lower their ethanol intake when paired with a low drinking peer, and these subpopulations should be explored for testing the efficacy of treatments to decrease ethanol use in groups that are likely to be responsive to different types of therapy.

## INTRODUCTION

Excessive alcohol (ethanol) use in the United States contributes to over 80,000 deaths per year (apps.nccd.cdc.gov/DACH_ARDI). Therefore, it is extremely important to understand all factors contributing to excessive ethanol drinking, as well as those that contribute to decreases in drinking. Peer influences can lead to increases in ethanol drinking in some cases, and to decreases in others. Both types of influence can be crucial on the path to either alcohol abuse (Fisher et al., 2007; Park et al., 2008) or abstinence (Gordon and Zrull, 1991; Bond et al., 2003; Wu and Witkiewitz, 2008; Kelly et al., 2011). Understanding the processes by which peer influences take effect will help inform and improve prevention and treatment strategies for alcoholism.

Biological mechanisms underlying peer influence are underexplored, in large part because such influence is difficult to model in laboratory animals. Most laboratory animals do not develop selective affiliations between individual adult animals and therefore cannot model specific social interactions between peers. In contrast, individuals of socially monogamous species do form such selective affiliations. For example, socially monogamous prairie voles (*Microtus ochrogaster*) exhibit increased preference not only for their sexual partner, but also to their same-sex cage mates (Getz et al., 1981; Williams, 1992; DeVries et al., 1997a). We have previously modeled specific social influences of ethanol drinking in prairie voles. Specifically, we have shown that, depending on the experimental conditions, housing with siblings or peers can either facilitate (Anacker et al., 2011a) or inhibit ethanol drinking in these animals (Anacker et al., 2011b). Moreover, such influence on ethanol drinking is specific to same-sex peers, and not male–female pairs (Hostetler et al., 2012).

The positive (inhibitory) influence of voles drinking low doses of ethanol on voles drinking high doses of ethanol was specific to ethanol, and was not observed with other palatable fluids (Anacker et al., 2011b). The decrease in ethanol drinking did not occur when high drinking animals were housed together, indicating that the high drinkers did not decrease their intake spontaneously or due to potential anxiety associated with cohabitation, but did so because of the influence of low drinkers. Moreover, the change in intake due to this peer influence was long-lasting and maintained even after the voles were separated. However, we also observed that while some of the voles changed their drinking behaviors due to influence of their peer, others did not. It is important to understand what makes a specific individual susceptible or resistant to peer influence, in order to target prevention or treatment accordingly. Based on our previous findings showing that high and low drinkers will alter alcohol intake levels when paired together, while matched drinkers will not, here we explored the manner in which the high–low drinking pairs affect one another. We hypothesized that high drinkers’ decrease in ethanol intake would be due to the development of a drinking pattern that was linked to that of a low drinking peer when they were housed together. To address this hypothesis here, we investigated features of prairie voles’ drinking patterns using a lickometer system.

Reports from other laboratories have demonstrated that the establishment of social bonds in prairie voles is dependent on the neuropeptide arginine vasopressin, acting via the vasopressin 1a receptor (V1aR; Winslow et al., 1993; Carter et al., 1995; Liu et al., 2001; Nair and Young, 2006; Donaldson et al., 2010). The gene for this receptor in prairie voles (*avpr1a*) contains a microsatellite region upstream of the transcription start site, which is polymorphic (Young et al., 1997; Hammock and Young, 2002, 2004; Hammock et al., 2005; Ophir et al., 2008; Solomon et al., 2009). Studies have demonstrated that the length of the microsatellite influences gene expression and receptor levels in many brain regions, and the expression in turn affects behavior (Hammock et al., 2005; Solomon et al., 2009). Specifically, several types of social behaviors including partner preference have been correlated with microsatellite length. In addition to vasopressin’s involvement in social behaviors, the neuropeptide levels are also affected by ethanol drinking and thought to play a role in the development of tolerance (Linkola et al., 1978; Hoffman et al., 1990; Inder et al., 1995; Harding et al., 1996; Rivier and Lee, 1996; Madeira and Paula-Barbosa, 1999; Silva et al., 2002). In addition, while one laboratory reported no effects of *avpr1a* deletion on ethanol intake (Caldwell et al., 2006), a more recent study found increased ethanol intake in *avpr1a* knockout mice (Sanbe et al., 2008). While studies on the role of *avpr1a *in alcohol drinking provided conflicting results, we explored whether the microsatellite length could relate to the degree of social influence on alcohol intake. Therefore, we further hypothesized that the length of the V1aR microsatellite could be correlated with ethanol drinking or the degree of social influence on ethanol drinking in prairie voles, and addressed this hypothesis in this study.

## MATERIALS AND METHODS

### ANIMALS

Prairie voles were bred in our colony at the Portland Veterans Affairs Medical Center Veterinary Medical Unit. All procedures were approved by the Institutional Animal Care and Use Committee and adhered to the guidelines put forth in the National Institutes of Health Guide for the Care and Use of Laboratory Animals. Voles were weaned around 21 days of age and housed in same-sex sibling pairs, with females and males housed in different rooms, until beginning the experiment. Voles were housed under 14L:10D lighting conditions and had continuous *ad libitum* access to water and food (corn, oats, and rabbit chow). Adult male and female voles (*n* = 95) were used in these studies, ranging from 58 to 95 days of age at the start of the experiment.

### APPARATUS

**FIGURE 1:**
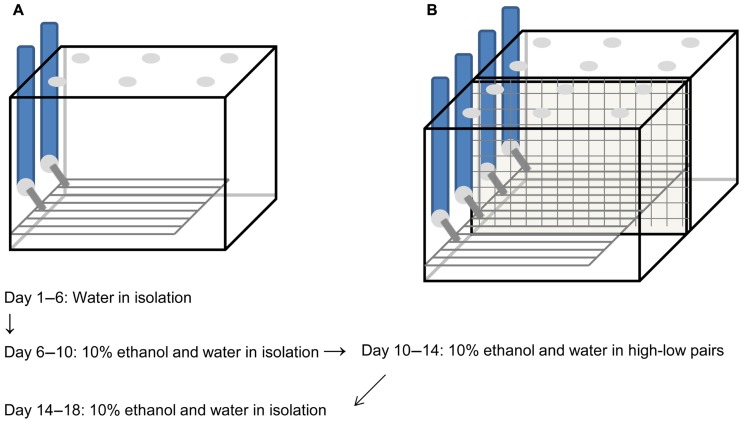
**Schematic diagram of lickometer cages and timeline.**Custom-designed cages were made to house voles individually **(A)** or in pairs separated by a mesh divider **(B)**. In both cases, plastic cages with air holes in the top surrounded wire metal racks that covered most of the cage floor. Voles had to step on the wire rack to reach the metal sipper tubes to obtain fluid, completing an electrical circuit to register each lick on the drinking tube. Subjects habituated to drinking water from the tubes for 5 days prior to the start of the experiment. They then had access to 10% ethanol and water for 4 days, after which time they were categorized as high or low drinkers. High and low drinkers were paired and given access to ethanol and water for another 4 days, followed by a final 4 days of isolation with continued access to ethanol and water.

The “lickometer” apparatus used in these experiments was a variation of that described previously (Ford et al., 2005; Anacker et al., 2011a). As before, the apparatus consisted of a metal floor (10 cm × 20 cm and 30 mm high; VWR, Tualatin, WA, USA), connected by electrical wires to metal spouts of the drinking tubes to create an open circuit through a dual lickometer device (MED Associates, Inc., St. Albans, VT, USA), which was connected to a PC. The wire bottom was positioned underneath the sipper tubes so that the animals were required to have at least one paw on the metal rack to touch the drinking spout, thereby completing the electrical circuit. Each lick exhibited by a subject was recorded by MED-PC IV software (MED Associates, Inc.) and stored for later analysis. The cage containing each apparatus was modified from the apparatus designed by Ford et al. and a schematic diagram is pictured in **Figure 1**. The plastic cage bottom that surrounded the wire rack was 16.8 cm × 27.6 cm and 5.4 cm high (Flair Plastic Products, Inc., Portland, OR, USA) and had bedding, food, and a nestlet available, so the subjects were not required to be on the wire rack when they were not drinking. The plastic cage top was 17 cm high and in addition to the holes for the drinking spouts, there were holes in the lid and openings along the bottom for air circulation (Flair Plastic Products, Inc.). The cages used for pair housing were identical except that they were twice as wide, with separate lids for each half, and a wire mesh down the center that divided the cage into two equal compartments but allowed the subjects visual, olfactory, vocal, and some tactile contact, similar to what has been described by us previously (Anacker et al., 2011a,b; Hostetler et al., 2012). Wire dividers were distant from the wire racks and drinking spouts and did not interfere with lickometer data collection.

### PROCEDURE

At the beginning of the experiment, voles were placed in individual lickometer cages and given access to water in the drinking tubes for 5 days, to habituate to the apparatus. After habituation, subjects were presented with ethanol in one drinking tube (10% ethanol by volume in tap water) and water in the other, and they had continuous access to these solutions throughout the rest of the experiment. Fluid volumes were recorded every 24 h, and the position of the bottles relative to one another was counterbalanced across pairs and switched every 2 days. Fluids were replaced every 2 days. After recording fluid volumes each day, and changing fluids every second day, the lickometer recording began and continued for 22 h.

After 4 days of access to ethanol in isolation, subjects were categorized as consistent high, medium, or low drinkers, dependent on the amount of ethanol they consumed (g/kg/day) and the preference ratio for ethanol over water, using identical criteria to a previous study (Anacker et al., 2011b). Specifically, high drinking was defined as no less than 9 g/kg of ethanol per day and no less than 0.75 ethanol preference over water. Low drinking was defined as less than 5 kg/day and less than 0.5 ethanol preference. After 4 days of baseline drinking in isolation, each animal was categorized by subtracting the number of “low” scores for preference and dose from the number of “high” scores. Animals receiving a positive number were labeled “high drinkers” while those receiving negative numbers were labeled “low drinkers.” Also as in other studies (Anacker et al., 2011b; Hostetler et al., 2012), high drinkers were paired with low drinkers and moved into the double cages with mesh dividers, where continuous access to ethanol and water continued for 4 days. Here, pairs were made up of same-sex, unrelated strangers. After pairing, subjects were again moved into isolation and had access to ethanol and water for a final 4 days. In this experiment, the controls similar to those used in past studies (namely high–high and low–low matched drinking pairs) were not used, since the focus of the study was on the behavioral mechanism by which the change in drinking occurs specifically in high–low pairs. Instead, subjects for comparison were generated based on individual performance in the experiment: subjects that changed their drinking level when paired were compared with those subjects that did not alter drinking.

Following the final isolation period, voles were euthanized by CO_2_ inhalation, and tail tissue samples were taken for genetic analysis.

### DRINKING ANALYSES

Ethanol intake and preference were calculated for each day based on fluid volumes consumed. Average measures for each housing period were compared by two-way repeated measures ANOVA with high and low drinkers as a between-subjects variable. Further analyses were done by splitting high drinkers into a group of animals that decreased their drinking level category during the 4 days of pair housing with a low drinker and a group of animals that did not, and comparing ethanol intake on each day of isolation and pair housing. Drinking data from days two and six are not presented in order to correspond with the lickometer data (see below), but data from each of these days were very consistent with the respective surrounding days. Bonferroni post-tests were used to determine specific group differences. As in a previous study (Anacker et al., 2011b), there were no sex differences in measures of alcohol consumption or the effects of pair housing on ethanol consumption and so data are presented and analyzed collapsed across sexes.

To validate the lickometer, water and ethanol volume consumed were each compared with the number of licks registered for each subject, and analyzed using a Pearson’s correlation.

The lickometer data were analyzed as described previously (Ford et al., 2005) by custom software for bout frequency (number of bouts), bout size, interbout interval, bout length, lick rate, and latency to first bout. For voles with zero or one drinking bouts per day, the data could not be analyzed using this software. However, the number of bouts for these subjects was included in the group analysis. Averages were compared by repeated measures ANOVA with three groups (high drinkers that remained high, high drinkers that decreased drinking level when paired, and low drinkers) as a between-subjects variable and each day throughout isolation and pair housing as the repeated measure. Due to a power failure, lickometer data for days two and six were not collected for a subset of animals. Rather than eliminating these subjects from the entire repeated-measures analysis, those 2 days were removed. Bonferroni post-tests were used to determine specific group differences.

The lickometer data were then processed using custom-designed software (u2615, Portland, OR, USA) which first rescaled the data from 10 ms to 1 s resolution. Cumulative lick plots for each pair on the last day of isolation and pairing were examined, since the subjects would have had the most time to establish stable drinking patterns under each housing condition. The number of bouts occurring in temporal proximity (≤15 min apart) was determined using a standardized visual assessment. The number of close bouts, and the number of close bouts normalized to the lowest number of bouts exhibited by one member of the pair, were compared using two-way repeated measures ANOVA with change in drinking as a between-subjects factor and housing as the repeated measure.

The data processed through u2615 were then analyzed for each pair by a cross-correlation analysis (R for Mac OS). The correlations were compared between the last day of isolation and the last day of pair housing. The presence or absence of a significant correlation for each day was noted, as well as the lag time and degree of correlation (autocorrelation function, ACF) for each significant correlation. The lag time range was limited to ±10 min, in order to analyze only behaviors that occurred close together in time. This metric indicated which subject followed the other in drinking, and was examined in conjunction with individual pair data indicating which subject changed intake.

### MICROSATELLITE LENGTH ANALYSIS

DNA was extracted from each subject’s tail tissue sample using a DNeasy Blood and Tissue Extraction Kit (Qiagen, Valencia, CA, USA). The V1aR microsatellite sequence was amplified using a variation of a previously published PCR technique (Hammock et al., 2005). We used the same sequences of primers but the forward primer was labeled with a 5-FAM fluorophore (Eurofins MWG Operon, Huntsville, AL, USA). We also used a touchdown PCR protocol to increase the specificity of the reaction (Korbie and Mattick, 2008), with a HotStarTaq DNA polymerase (Qiagen). The reactions were heated to 94°C for 15 min to activate the polymerase, and then had 28 cycles of 30 s denaturing (94°C), 45 s annealing, and 1 min for elongation (72°C). The annealing temperature started at 71°C on the first cycle and decreased by 1°C in each of the following 12 cycles. The last 25 cycles all had an annealing temperature of 58°C. The reaction was ended by a 5 min elongation at 72°C and cooling to 4°C.

The samples were each read by a 3130xl Genetic Analyzer (Applied Biosystems, Carlsbad, CA, USA), by the Oregon Clinical and Translational Research Institute Core Laboratory at Oregon Health & Science University (OHSU). The microsatellite length was determined for each allele for each subject with approximately 3 base pair resolution.

Microsatellite allele lengths were not normally distributed due to a highly leptokurtotic sample, which could not be normalized by any transformation. Thus, correlations could not be conducted using the collected data. Instead, a median split was applied to the data and *t*-tests were performed to compare between animals that had short or long average microsatellite length. A number of dependent variables were tested (baseline preference and intake, change in preference and intake for high or low drinkers between isolation 1 and pairing, or pairing and isolation 2, or overall from isolation 1 to isolation 2) and a Bonferroni correction for multiple comparisons was applied to yield the corrected threshold for significance α = 0.005.

## RESULTS

High drinkers were paired with low drinkers, leading to a total of 32 pairs that completed the experiment, while medium drinkers did not continue past the initial isolation phase. Of these, 14 pairs were female and 18 pairs were male.

**FIGURE 2:**
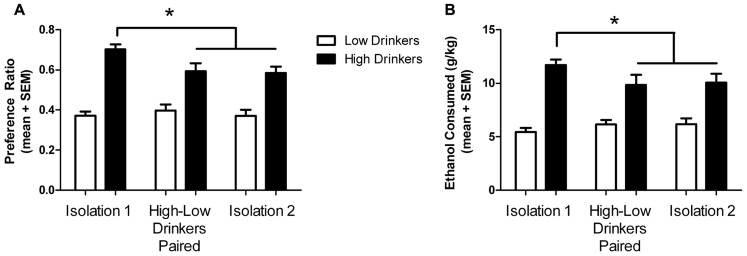
**Ethanol preference and intake in different housing conditions.** Ethanol preference (A) and intake **(B)** by high (black) and low (white) drinkers in each housing condition. *Significant difference between isolation 1 and subsequent housing conditions for high drinkers; *p* < 0.05.

As expected, high drinkers had a significantly higher preference for ethanol than low drinkers (high: 0.703 ± 0.024; low: 0.372 ± 0.030; *F*_(1,62)_ = 45.71; *p* <0.0001) and exhibited higher intakes (high: 11.7 ± 0.536 g/kg; low; 5.45 ± 0.369 g/kg; *F*_(1,62)_ = 40.85; *p* <0.0001), in accordance with their categorization. There was a significant effect of housing conditions on preference (*F*_(1,124)_ = 4.91; *p* = 0.009) but not intake (*F*_(1,124)_ = 0.82; *p* = 0.441). As seen in our previous study (Anacker et al., 2011b), there was a significant interaction between drinking category and housing condition on preference (*F*_(1,124)_ = 6.94; *p* = 0.0014) and intake (*F*_(1,124)_ = 4.48; *p* = 0.013). Planned Bonferroni post-tests revealed that high drinkers decreased their ethanol preference from baseline (isolation 1) to paired housing and isolation 2 (*t* = 3.93 and 3.26, respectively; df = 15; *p* < 0.001), as well as the intake level (*t* = 2.76 and 2.44, respectively; df = 15; *p* < 0.05), while low drinkers did not significantly change (**Figure 2**).

**FIGURE 3:**
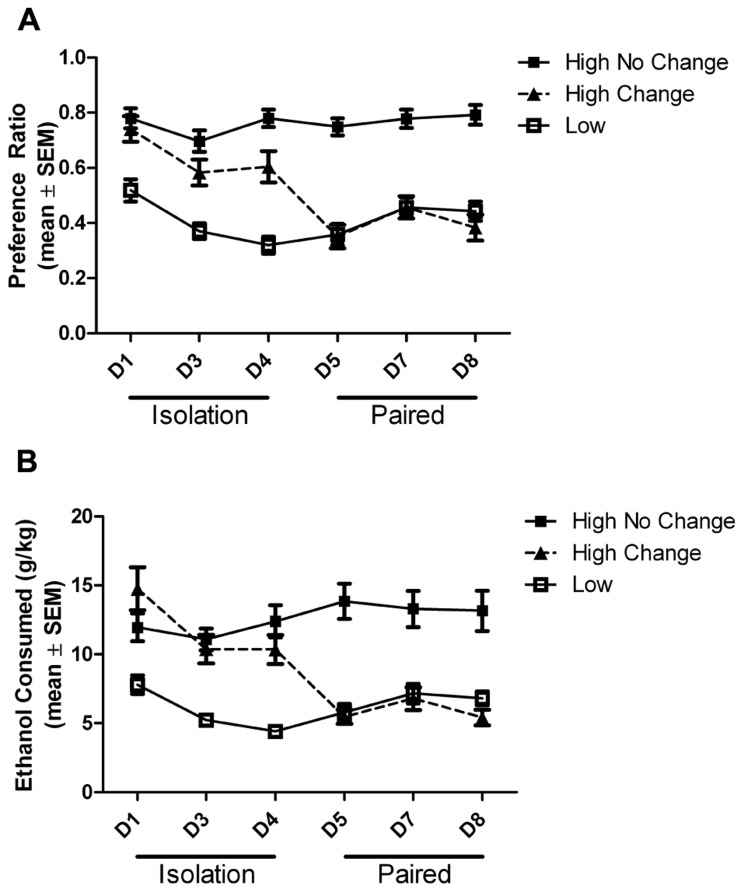
**Ethanol preference and intake across days.** Ethanol preference **(A)** and intake **(B)** by high drinkers that did (black square) or did not (black triangle) change ethanol intake when paired with low drinkers (white square) is shown across days of isolation and pair housing. In isolation, both high groups are significantly higher than the low drinkers. During pair housing, the high drinkers that change intake and the low drinkers are both significantly lower than the constant high drinkers.

Also as in the previous study (Anacker et al., 2011b), the behavior of individual animals within the high drinkers differed, and they could be subcategorized into animals that either did (15/32; ~47%; 7 female and 8 male) or did not (17/32; ~53%; 7 female and 10 male) change their drinking under social conditions. The change was defined as the subject’s average drinking during pair housing meeting the criteria for a drinking level different than the baseline drinking level. While all high drinkers had greater ethanol preference and intake than low drinkers on the first and last day of the first isolation period, only those high drinkers that altered their drinking under social conditions decreased their preference and intake to the level of the low drinkers during pair housing (**Figure 3**). There was a main effect of group on ethanol preference (*F*_(2,305__)_ = 51.65, *p* < 0.0001) and intake (*F*_(2,305__)_ = 34.47, *p* < 0.0001), a main effect of day on preference (*F*_(5,305__)_ = 10.26, *p* < 0.0001) and intake (*F*_(5,305__)_ = 7.66, *p* < 0.0001), and an interaction between the group and housing on preference (*F*_(__10__,305__)_ = 6.40, *p* < 0.0001) and intake (*F*_(10,305__)_ = 9.86, *p* < 0.0001). *Post hoc* tests revealed that the low drinkers had significantly lower ethanol preference and intake than both groups of high drinkers on each day of isolation (*p* < 0.001), while during pair housing, both the low drinkers and the high group that changed had significantly lower ethanol intake than the high drinkers that did not change (*p* < 0.001). On day 4 of isolation, the high-change group had a significantly lower preference for ethanol than the high-no change group (*p* < 0.05), while still remaining significantly higher than the low drinkers, as described above.

**FIGURE 4:**
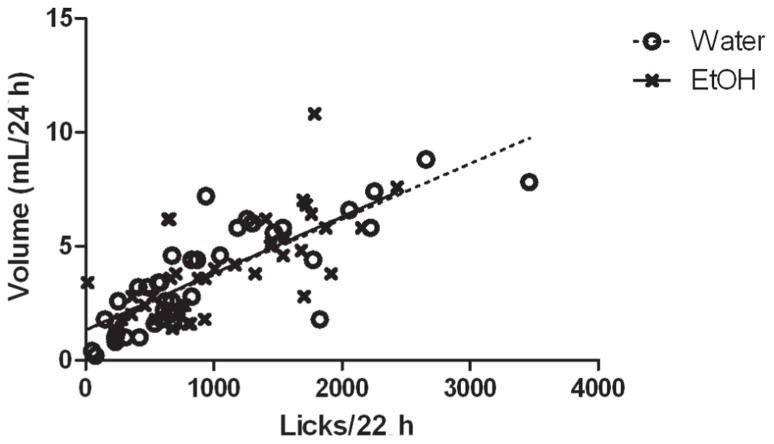
**Correlation of recorded licks with fluid volume consumed.** The relationship between the number of recorded licks from each drinking tube on the *X*-axis with the volume of water (O) or ethanol (X) consumed on the *Y*-axis, for each of 4 days in isolation is graphed for one cohort of animals (*n* = 10) representative of the entire experiment. There was a strong positive correlation for both water (*r* = 0.815, *n* = 40, *p* < 0.0001) and ethanol (*r* = 0.694, *n* = 40, *p* < 0.0001).

**FIGURE 5:**
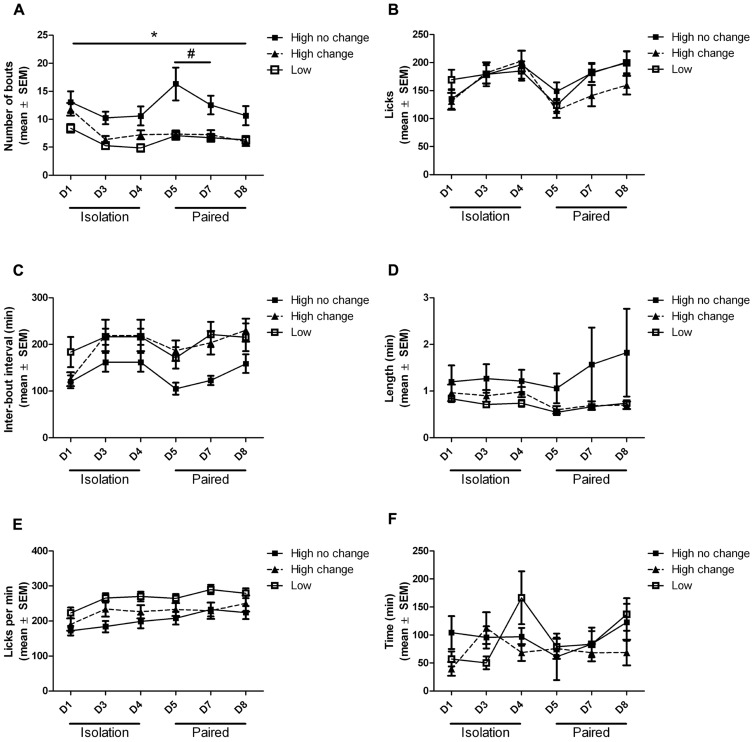
**Ethanol drinking bout features.** Bout features of high drinkers that did (black triangle) or did not (black square) change ethanol intake when paired with low drinkers (white square) are shown throughout isolation and pair housing. The number of bouts of ethanol drinking **(A)**, the average length of bouts as measured by number of licks **(B)** and time **(D)**, the average time between bouts **(C)**, the rate of licks within a bout **(E)**, and the average time until the first lick was recorded **(F)** are shown for each 22 h period. *Post hoc significant difference between high-no change group and low group; #*Post hoc* significant difference between high-no change group and high-change group.

Volumes of ethanol and water consumed each day correlated very well with the number of licks recorded for each subject (**Figure 4**). Analysis of the bouts of ethanol consumption revealed one notable difference between high drinkers that did not change ethanol intake when paired with low drinkers, and high drinkers that did change, out of six different parameters assessed (**Figure 5**). Since the software could not analyze data from subjects with one or fewer drinking bouts, 10 low drinkers and one high drinker were not included in the analysis of other features besides the number of bouts. There was a main effect of group on the number of bouts (*F*_(2,3__00)_ = 10.69; *p* = 0.0001), interbout interval (*F*_(2,24__0)_ = 5.71; *p* = 0.006), and lick rate (*F*_(2,225__)_ = 5.30; *p* = 0.009). There was also a main effect of day on the number of bouts (*F*_(5,300)_ = 12.82; *p* < 0.0001), interbout interval (*F*_(5__,__24__0)_ = 5.33; *p* = 0.0001), and lick rate (*F*_(5__,__225__)_ = 11.17; *p* < 0.0001). Most notably, there was an interaction effect of group and day on the number of bouts (*F*_(10,300)_ = 3.06; *p* = 0.001).

*Post hoc* tests revealed the source of the interaction between group and day on the number of ethanol drinking bouts. The number of bouts was significantly higher in the high-no change group than in the low group on all days (*p* < 0.05), while the high-change group was never significantly different from the low group. The high-change group did have significantly fewer drinking bouts than the high-no change group on days 5 (*p* < 0.001) and 7 (*p* < 0.05) during pair housing (**Figure 5A**).

**FIGURE 6:**
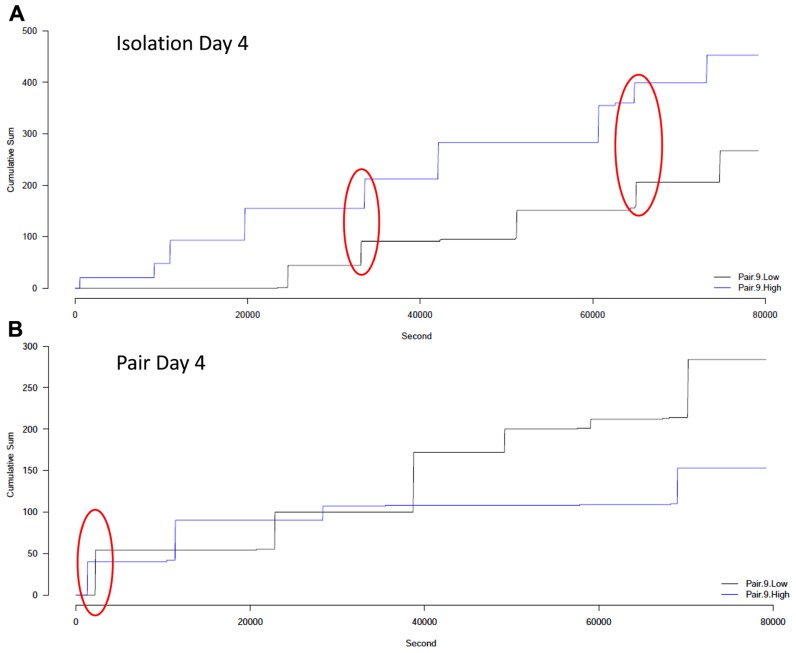
**Cumulative number of licks of ethanol over 22 h for an example pair. (A)** The drinking patterns for subjects in a pair on the last day of isolation. **(B)** The drinking patterns for subjects in the same pair on the last day of pair housing. The high drinker is shown in blue and the low drinker is shown in black. Each “step up” in the graph indicates a bout of drinking while each horizontal line indicates a time when no drinking occurred. The red circle indicates bouts that occurred close together in time, within the applied threshold.

**FIGURE 7:**
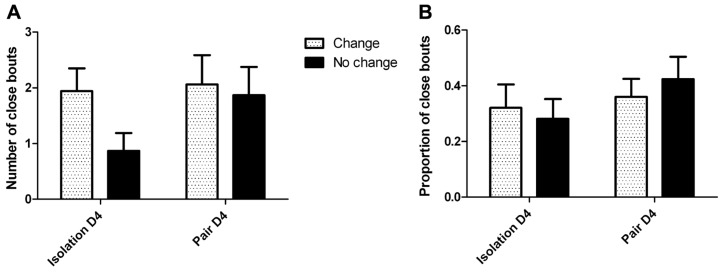
**Visual assessment of close ethanol drinking bouts between partners in isolation and pair housing.(A)** The number of close bouts does not significantly differ between housing conditions or group changes, and there is no significant interaction of effects. **(B)** The proportion of close bouts relative to the lowest number of bouts one subject exhibited does not significantly differ between housing conditions or group changes, and there is no significant interaction of effects.

Visual analysis of the cumulative lick graphs (**Figure 6**) revealed that while there were occurrences of ethanol drinking bouts close together in time for pairs of animals, the frequency of close bouts was not significantly different between isolation (**Figure 6A**) and pair housing (**Figure 6B**), or between pairs that did not change drinking levels compared to those who did, and there was no interaction between the two factors when either the number (**Figure 7A**) or proportion (**Figure 7B**) of close bouts was assessed.

**FIGURE 8:**
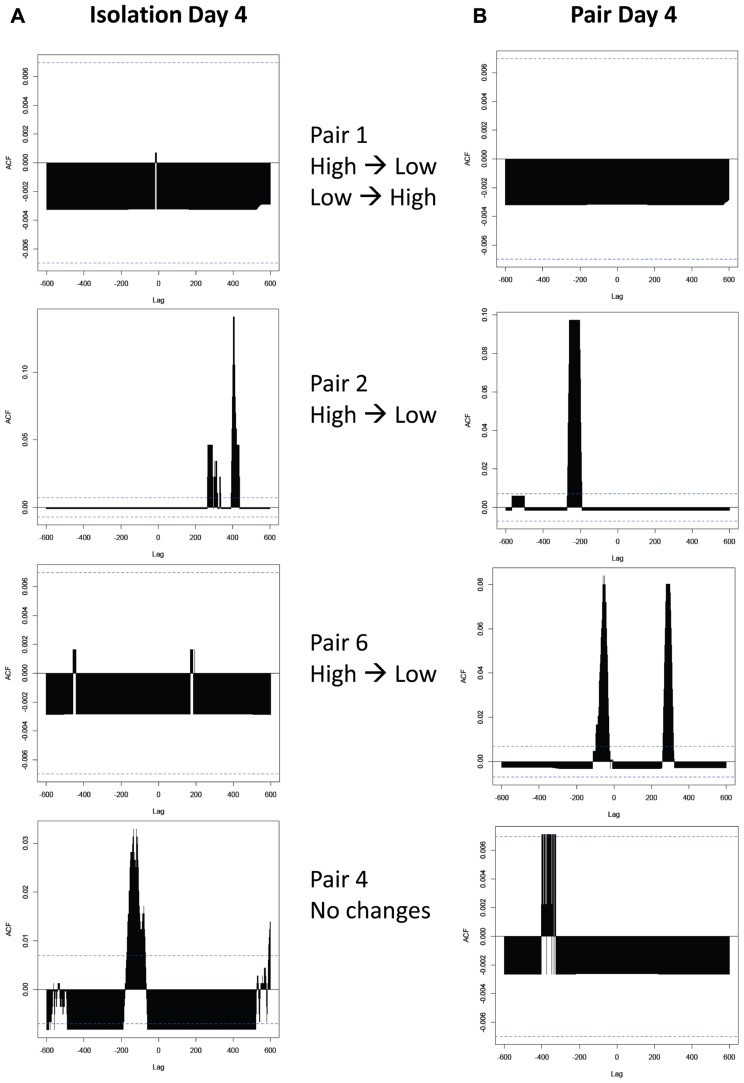
**Cross-correlations between ethanol drinking patterns of peers. (A)** Correlations between high and low drinkers in isolation, before they have been housed together. **(B)** Correlations between high and low drinkers in pairs on the fourth day of pair housing. The *Y*-axis represents the strength of the correlation (autocorrelation function; ACF), while horizontal dashed lines represent the threshold for significance. The *X*-axis represents “lag” time, in seconds, between drinking events. A significant correlation at a lag time “*h*” indicates that *h* seconds after the high drinker licks, the low drinker is likely to lick; a positive *h* value indicates that the high drinker leads the low drinker, and a negative value indicates that the low drinker leads the high drinker. Pairs shown are examples of each type of pair observed: those where both subjects changed drinking levels when paired (top panels), those where the high drinker changed to match the low drinker (middle panels show two of many variations of outcomes), and those where neither subject changes (bottom panels).

Cross-correlation analyses revealed that over two-thirds of the pairs exhibited a significant correlation between ethanol drinking patterns regardless of whether they were physically isolated (**Figure 8A**) or housed together (**Figure 8B**). Additionally, there was no consistent difference in the presence or absence of correlations between pairs that exhibited changes in drinking behavior and those that did not (**Table 1**). Contrary to our hypothesis, there was no consistent directionality of the lag time of cross-correlations in pairs that changed their drinking level: in pairs where high drinkers changed to low drinkers, there was not a greater presence of a negative lag time that would indicate the low drinker “leading” the high drinker (**Figure 8B** panels 2 and 3).

**Table 1:** Number of pairs exhibiting significant correlations in drinking patterns.

		Ethanol isolation 1	Ethanol pair	Water isolation 1	Water pair
Change	Correlation	11	12	14	14
	No correlation	6	5	3	3
No change	Correlation	12	12	12	15
	No correlation	3	3	3	0

*The total number of pairs (32) is divided into groups where at least one subject changed the drinking pattern and those that did not (change, no change). For each group, the number of pairs exhibiting a significant correlation or not, when analyzed by the cross-correlation function within a lag time of 10 min is shown for each ethanol and water drinking patterns, in isolation and pair housing. There are clearly no significant differences between groups that did or did not change, between isolation and pairing, or between ethanol and water.*

There was no significant correlation between the number of close bouts by visual assessment and the strength of cross-correlations (ACF value; *r* = 0.083; *n* = 19; *p* = 0.734). However, there was a statistical trend for a positive correlation between the *proportion* of close bouts by visual assessment (number normalized to the lowest number possible for each pair) and the strength of cross-correlations (*r* = 0.444; *n* = 18; *p* = 0.065).

The region containing the V1aR microsatellite was successfully amplified and lengths were determined for 59 subjects. The rate of homozygosity was 47%. The length of the amplified region ranged from 669 to 736 base pairs. The mean, median and mode for all alleles were 703, 699, and 698 bp, respectively. The allele lengths were not normally distributed.

There was no significant difference in drinking behavior between subjects with short or long average microsatellite lengths on any measure of behavior (**Table 2**): initial ethanol preference, initial ethanol intake, change in ethanol preference or intake from isolation to pair housing, pair housing to subsequent isolation, or overall change from the beginning to the end of the experiment. There was a difference within high drinkers, where subjects with long alleles had a greater decrease in ethanol preference from the beginning to the end of the experiment than those with short alleles (*t* = 2.27; df = 26; *p* = 0.031), but this difference did not remain significant when adjusted for multiple comparisons.

**Table 2:** Effect of V1aR microsatellite length on ethanol drinking behaviors.

Behavior	*t*,df	*p* Value
Baseline preference	*t* = 0.151 df = 57	0.880
Baseline intake	*t* = 1.37 df = 57	0.176
High preference change 1	*t* = 1.30 df = 26	0.207
High preference change overall	*t* = 2.27 df = 26	**0.0314**
Low preference change 1	*t* = 0.257 df = 29	0.799
Low preference change overall	*t* = 0.638 df = 29	0.529
High intake change 1	*t* = 0.166 df = 26	0.870
High intake change overall	*t* = 0.441 df = 26	0.663
Low intake change 1	*t* = 0.000571 df = 29	1.00
Low intake change overall	*t* = 0.0418 df = 29	0.967

*Subjects possessing a short or long average microsatellite length were compared for each of the behaviors listed in the column on the left. The results of the t tests are listed in the middle column, with the *p* value in the right. Significant (*p* < 0.05) values prior to correction are shown in bold, though no tests remained significant when the Bonferroni correction was applied (*p* < 0.005). Baseline preference and intake were based on the average ethanol (and water) intakes of the first 4 days of access, during isolation. “High” indicates a comparison between only the high drinkers, and “Low” indicates only the low drinkers. “change 1” indicates the change in drinking from the average during isolation 1 to the average during pair housing. “change overall” indicates the change in drinking from the average during isolation 1 to the average during isolation 2 following pair housing.*

## DISCUSSION

Prairie voles drinking large amounts of ethanol paired with low drinkers in the lickometer apparatus exhibit a decrease in drinking similar to what we have previously demonstrated in home cage drinking (Anacker et al., 2011b). This previous study has already indicated that this decrease is not spontaneous but is due to social influence. The present experiments indicate that the observed changes in ethanol drinking are not dependent on peers drinking together at the same time, or following specific patterns of consumption. Accordingly, this finding is in agreement with our previous results which showed that no changes in saccharin drinking occurred when high drinkers were paired with low drinkers (Anacker et al., 2011b). Specifically, the lack of changes in saccharin drinking suggested that even if pair housing of animals could synchronize their consummatory behaviors, this synchronization is not sufficient to affect their individual drinking levels. However, there are subtle differences in the features of voles ’ drinking bouts that can differentiate which subjects change their intake when paired. Specifically, high drinkers that lowered their ethanol intake when paired with a low drinker exhibited a lower number of ethanol drinking bouts when paired than the high drinkers that did not change. It is interesting to note that a tendency toward a lower number of drinking bouts was present in this group even before pairing, along with gradual decreases in intake and preference across days in isolation. The high-change group showed a tendency toward a lower number of ethanol drinking bouts in isolation relative to the high drinking group, while the intake levels remained similar; this may be explained by the slight increase in the lick rate of the high-change group, which would allow them to maintain the high level of intake while decreasing the number of bouts. The high-change group also exhibited a tendency toward higher total fluid consumption relative to the high-no change group, resulting in a similar ethanol dose consumed but a lower preference in the high-change group compared to the high-no change group. This observation suggests that the pairing with a low drinker interacted with this tendency toward lower preference and lower number of bouts to produce a more robust decrease in ethanol intake. Thus, we could differentiate subpopulations of high drinkers that were and were not responsive to social influence to decrease ethanol intake, based on differences in fluid preference and on the number of drinking bouts that already existed when isolated.

These findings have potential to lead to future translational work. It is widely known that different types of therapies work for only subsets of people with alcohol use disorders (Anton et al., 2006). Some people may be responsive to social support groups, others to drug therapies, and others to cognitive or behavioral therapy, while still others benefit from a combination. It would be extremely helpful if there were tools to allow clinicians to identify these subpopulations in order to target appropriate treatment to achieve the greatest effect to decrease problem drinking. To our knowledge, there is currently no other animal model where subpopulations that are likely to be responsive to different types of treatments have been identified. If further studies identified behavioral and biological mechanisms of actions or endophenotypes that could predict the success of social influence on lowering drinking, this information could be explored to improve treatment outcomes. Future studies could also test whether the group of high drinking voles that was unresponsive to pairing with a low drinker would be more responsive to pharmacotherapy than to peer influence to decrease drinking.

Furthermore, it remains to be explored why a subset of voles was susceptible to peer influence. One possibility is that different levels of anxiety predispose particular individuals to imitate or avoid a peer. The argument could be made in either direction: higher social anxiety could lead to an increase in trying to “blend in ” or to avoid contact and influence from a peer. Hostetler et al. (2012) showed that baseline anxiety-related behavior in the elevated plus maze was correlated with alcohol drinking, although the correlation was higher in isolated housing than in paired housing, and specifically in males, but it remains possible that individual differences in baseline or reactive anxiety are associated with the changes in alcohol drinking levels. While no measures of stress or anxiety levels were taken in the current study, it needs to be noted that early studies did not find effects of same-sex pairing on glucocorticoid levels (DeVries et al., 1997b). Since the initial aim of this study was to examine drinking patterns of behavior without disturbing the animals, future studies should examine different types of anxiety in relation to alcohol intake, as well as corticosterone levels. It is also possible that the voles that responded to peer influence have different social behaviors overall, which may have led them to alter their drinking behavior, for example to spend more time interacting with the partner rather than drinking. It would be interesting to explore social behaviors, e.g., in a social interaction test, to examine how they relate to propensity to alter drinking behavior in a social context.

Interestingly, specific episodes of peer influence were not detected by any comparisons of drinking patterns undertaken here. The visual assessment of the cumulative lick records and the cross-correlation analyses both indicated that subjects often have drinking bouts that are close together in time. We initially hypothesized that these coincident drinking bouts would occur more often when pairs were housed together than when they were in isolation, since they may synchronize their ultradian rhythms to be awake and feeding and drinking at the same time. However, this was not the case; nearly equal numbers of pairs had significant correlations in isolation and in paired housing.

While we found that neither cumulative lick record nor cross-correlation analyses revealed evidence of consistent patterns of linked ethanol intake in pairs, we also found that these different analyses did not exhibit strong correlations with one another. In particular, we would have expected a large number of close drinking bouts in a visual assessment of drinking patterns to be associated with a stronger ACF value in the cross-correlation, but this positive correlation did not reach statistical significance. There are many possible reasons for this. One explanation is that the lag time between bouts would have to be nearly identical within a pair in order to produce a strong ACF by cross-correlation. If the time between paired subjects ’ drinking bouts varied even by 30 s for each bout, it is possible that a significant ACF value would never be produced by cross-correlation: each lag time would be cataloged, but would have such a low frequency of occurrence that none would be considered significant. In this case, with animal behavior having the potential to be extremely variable even within a framework of a consistent pattern, cross-correlational analyses may not be optimal for detecting such patterns.

Given the evidence from the various types of pattern analyses performed in this study, it appears that prairie voles do not alter their ethanol drinking behavior by synchronizing their drinking patterns with those of a peer. Therefore, another mechanism must be at work to explain the peer-dependent change in drinking levels observed in the present study and previous work, where most often the high drinker decreases its intake when paired with a low drinker. Thus, it is an open question whether the low drinker is typically the dominant vole within the pair and, if so, how this may dictate ethanol intake or changes in ethanol intake. Another possible explanation is that the voles try to match one another ’s intoxication levels, perhaps through visual cues or vocal interactions. This explanation would address the specificity of behavioral changes observed for ethanol but not saccharin, a rewarding substance that does not lead to intoxication.

The length of the vasopressin receptor 1a (*avpr1a*) microsatellite fragment observed here was different than what has previously been reported by others. Hammock and Young (2005) and Solomon et al. (2009) reported a range of 723 –760 and 703 –798 base pairs, respectively, which are considerably longer and show very little overlap with our sample. Additionally, they observed between 75 and 100% heterozygosity and a normal distribution while almost half or our sample was homozygous, leading in part to a highly leptokurtic distribution. Since the subjects in our study arose from different colonies of prairie voles than the previously published findings, it is possible that they originate from a different subsample of the wild prairie vole population, and that in our colony we have a larger presence of similarly sized alleles leading to a higher frequency of particular alleles and homozygosity.

 In addition to differences in allele length in the samples, the present experiment did not find effects of the microsatellite length on any measure of prairie voles ’ ethanol drinking behavior, or on the propensity to change ethanol intake when paired with a peer. There was an indication of an effect of the longer microsatellite length corresponding to a greater change in ethanol preference following the effect of a peer influence, but this effect did not remain significant following adjustment for multiple comparisons. Thus, this trend should only be considered suggestive of the potential for the *avpr1a* microsatellite to modulate social influence on ethanol drinking. A recent study in human adolescents demonstrated no role of a different repeat region, a variable number tandem repeat in the dopamine D4 receptor gene, in the effects of friends ’ drinking levels on subjects ’ drinking (van der Zwaluw et al., 2012).

The effects of the V1aR microsatellite length reported by others appear to be very specific to particular tests and environments. For example, microsatellite length was correlated with the receptor expression level in various brain regions, and several of these regions were then correlated with measures of partner preference in the laboratory test (Hammock et al., 2005), but not when laboratory-bred voles were tested for social monogamy in semi-natural enclosures (Ophir et al., 2008; Solomon et al., 2009), or in wild prairie voles (Mabry et al., 2011). In contrast, the length was correlated with genetic monogamy in the wild, but not in semi-natural enclosures.

One possible reason for effects that may be difficult to detect has previously been proposed by others (Ophir et al., 2008): while there are several ways in which microsatellite length may influence expression levels (Hammock and Young, 2005), it is likely that particular single nucleotide polymorphisms in *avpr1*, rather than its length, could be a better predictor gene expression and, ultimately, behavior.

## CONCLUSION

The present study shows that while high drinkers decrease their ethanol intake when paired with low drinkers, it is not due to matching patterns of drinking, and the behavioral changes cannot be predicted by the length of the microsatellite polymorphism in the vasopressin receptor 1a. Other behaviors and specific genetic polymorphisms that may affect peer-influenced ethanol drinking may be studied in the future. This study demonstrates new methods for examining data from fluid consumption studies where social influences can be assessed using visual and cross-correlational analyses. Most importantly, this study shows that subpopulations of high drinkers that decrease their ethanol intake can be identified based on changes in intake levels and bout number when paired with a low drinker. This provides a model system in which the efficacy of potential therapies can be tested using groups which are likely to respond to different types of treatments. It will be important to examine whether subpopulations of human alcohol drinkers can be identified with similar means, and to explore whether they are similarly responsive to social or other types of treatments to decrease alcohol drinking; then further testing of this animal model of alcohol drinking can be used to elucidate specific mechanisms of action and responses to treatments that can inform treatment of humans with alcohol use disorders.

## Conflict of Interest Statement

The authors declare that the research was conducted in the absence of any commercial or financial relationships that could be construed as a potential conflict of interest.
